# A Review of the Structure and Physical Properties of Fluorozirconate and Rare-Earth-Doped ZBLAN Glasses

**DOI:** 10.3390/ma19163511

**Published:** 2026-08-19

**Authors:** Pantelis Mpourazanis, Christelle Kielleck, Marc Eichhorn

**Affiliations:** 1 Fraunhofer Institute of Optronics, System Technologies and Image Exploitation, 76275 Ettlingen, Germany; christelle.kieleck@iosb.fraunhofer.de (C.K.); marc.eichhorn@iosb.fraunhofer.de (M.E.); 2 Institute of Control Systems, Karlsruhe Institute of Technology, 76131 Karlsruhe, Germany

**Keywords:** fluorozirconate glasses, ZBLAN glass, rare-earth-doped glasses, structure-property relationships

## Abstract

Heavy metal fluoride glasses (HMFGs), particularly fluorozirconate glass systems such as ZBLAN have attracted considerable attention due to their unique physical properties, including low phonon energies, wide transparency from the UV to the mid-IR, and high rare-earth ion doping solubility, making them promising materials for photonic applications. This review provides an overview of fluoride glass synthesis methods, structural characteristics, and physical properties of fluorozirconate glasses, with emphasis on glass processing conditions, thermal, mechanical, and optical properties. The structural characteristics are discussed in terms of zirconium–fluorine polyhedral networks and their compositional dependence, while physical properties are analyzed, including glass transition behavior, crystallization tendency, elastic moduli, and infrared transmission. Rare-earth doped Er^3+^, Ho^3+^, and Tm^3+^ ZBLAN glasses are also discussed, which exhibit efficient emissions in the near and mid-IR spectral regions. Although significant progress has been achieved, limitations related to thermal stability, mechanical strength, and incomplete understanding of structure–property relationships persist. Future research should therefore focus on compositional optimization and predictive structural modeling to enable the design of improved fluoride glasses for various applications.

## 1. Introduction

Heavy metal fluoride glasses (HMFGs) have attracted significant interest in the glass science community since the discovery of fluorozirconate glasses by the Poulain brothers in 1974 at the University of Rennes in France [1]. Since then, several multicomponent HMFG compositions have been synthesized and investigated for their physicochemical properties.

Based on the primary glass former, these materials are commonly classified into fluorozirconates (ZrF_4_-based), fluoroaluminates (AlF_3_-based) and fluoroindates (InF_3_-based). While common oxide glasses such as silicates, phosphates, and tellurites exhibit physicochemical properties tailored by their specific structure, fluoride glasses possess fundamentally different structural configurations that yield distinct physical and chemical properties. Moreover, HMFGs require different glass processing methods compared to oxide glasses because they exhibit a strong tendency toward crystallization during melting and cooling, arising from their low viscosity values near the melting point [2]. Fluoride glasses exhibit low phonon energies and high optical transparency from the ultraviolet to the mid-infrared region. These characteristics suppress multiphonon relaxation of excited rare-earth ions, enabling efficient radiative transitions and making them attractive host materials for active optical devices [3,4,5]. Specifically, their high infrared transparency (2–7 μm), high rare-earth solubility, and low attenuation make fluoride glasses excellent candidates for mid-IR optical fibers, fiber lasers, and supercontinuum generation, where conventional silicate and phosphate fibers exhibit significantly higher transmission losses [6,7,8].

Among fluorozirconate glasses, the most widely studied composition is the multicomponent system 53ZrF_4_-20BaF_2_-4LaF_3_-3AlF_3_-20NaF, (mol%), commonly referred to as ZBLAN glass [1,9]. The acronym ZBLAN originates from the main glass constituents: zirconium, barium, lanthanum, aluminum, and sodium fluorides. ZBLAN is considered the most crystallization-resistant heavy metal fluoride glass and is therefore commonly used for optical fiber drawing. Early theoretical studies predicted that the minimum optical transmission loss for fluoride glass fibers could reach approximately 10^−3^ dB/km at wavelengths around 3.5 μm [10]. Subsequent experimental investigations estimated the minimum optical loss coefficient of ZBLAN glass to be on the order of 10^−2^ dB/km at 2.5 μm [11,12]. Although the theoretical minimum attenuation of ZBLAN fibers has been predicted to approach 10^−3^–10^−2^ dB/km in the 2.5–3.5 μm spectral region, practical commercial fibers typically exhibit background losses below 10–50 dB/km because of extrinsic absorption and scattering arising from impurities, structural defects, and partial crystallization introduced during glass synthesis and fiber drawing [5,13,14]. By comparison, state-of-the-art silica optical fibers exhibit ultra-low losses of approximately 0.14–0.17 dB/km at 1.55 μm, but their transmission rapidly deteriorates beyond ~2.2 μm due to multiphonon absorption, while phosphate and tellurite glasses also exhibit substantially higher mid-infrared losses than fluoride fibers [15,16]. Consequently, ZBLAN remains one of the most attractive fiber materials for low-loss transmission and laser applications in the 2–5 μm spectral region, motivating continued efforts to improve its thermal stability, chemical durability, and resistance to crystallization through compositional optimization and advanced glass processing techniques.

Among rare-earth doped systems, Er^3+^, Ho^3+^, and Tm^3+^ doped ZBLAN glasses have attracted particular attention because of their efficient mid-infrared emissions. Er^3+^ doped ZBLAN glasses exhibit emission bands near 1.55 and 2.7 μm, originating from the ^4^I_13/2_ → ^4^I_15/2_ and ^4^I_11/2_ → ^4^I_13/2_ transitions respectively [17,18,19]. Applications of Er^3+^ doped ZBLAN glasses include optical sensing, fiber lasers, light detection etc. [20,21]. Furthermore, Er^3+^-doped fluorozirconate glasses like ZBYA have been reported to exhibit improved thermal stability compared to Er^3+^ doped ZBLAN glass, while maintaining spectroscopic properties suitable for applications in infrared lasers and optical amplifiers [22].

Ho^3+^ doped ZBLAN glasses also exhibit promising spectroscopic characteristics for laser emissions in the near and mid-IR regions. Emission bands around 1.2, 2.1 and 2.9 μm have been reported, corresponding to the ^5^I_6_ → ^5^I_8_, ^5^I_7_ → ^5^I_8_ and ^5^I_6_ → ^5^I_7_ transitions, respectively [23]. These transitions enable the development of fiber lasers operating near 1.2 μm and 2 μm based on Ho^3+^ doped fluorozirconate glass fibers. Similarly, Tm^3+^ doped ZBLAN glasses are characterized by an emission near 1.8–2 μm spectral region, arising from the ^3^F_4_ → ^3^H_6_ transition, attributed to the inhomogeneous distribution of Tm^3+^ ions within the glass network [24,25]. The relatively low phonon energy of fluoride hosts suppresses non-radiative decay, thereby enabling efficient radiative transitions that are often quenched in oxide-based glasses [26]. Tm^3+^ doped fluoride glasses exhibit strong emission in the 1.8 μm spectral region under ~800 nm excitation, corresponding to the ^3^F_4_ → ^3^H_6_ transition, which is significantly enhanced by cross-relaxation processes between Tm^3+^ ions [16]. These favorable spectroscopic properties make Tm^3+^ doped fluoride glasses promising candidates for fiber laser applications and multi-band infrared sources [16,26].

In this review, we summarize results from key studies on the synthesis, structure, and physical properties of fluorozirconate glasses, with a particular focus on ZBLAN glass. After discussing the fundamental properties of fluorozirconate glasses, we examine the spectroscopic properties of Er^3+^, Ho^3+^, and Tm^3+^ doped ZBLAN glasses, which are among the most extensively investigated rare-earth systems for near and mid-infrared photonic applications. Given the large number of fluoride glass-forming systems and rare-earth dopants reported in the literature, a comprehensive review of all fluorozirconate glass compositions is not feasible. Instead, we highlight representative studies that have made significant contributions to understanding the structure–property relationships of fluorozirconate glasses. Although ZBLAN glasses are widely employed as hosts for infrared optical fibers, the present review focuses on the fundamental physical, structural, and spectroscopic properties of bulk fluorozirconate glasses. Therefore, topics such as optical fiber fabrication, industrial-scale production, and commercialization are beyond the scope of this review. Nevertheless, understanding the properties of bulk fluoride glasses provides the scientific foundation for the development of next-generation mid-IR photonic devices and fluoride fiber laser technologies.

## 2. Synthesis and Preparation of Fluorozirconate Glasses

The synthesis and preparation of high-quality fluoride glasses require raw materials of high purity, containing minimal concentrations of impurities such as water and transition metals. The formation of unwanted crystallites significantly compromises the optical quality of the glass by introducing surface defects and increasing scattering losses, thereby degrading performance [27]. Hence, strict control of atmospheric conditions and precursor purity is essential to prevent the formation of oxyfluorides and to minimize crystallization and associated optical losses [28,29].

To solve such issues arising from water and other contaminants, pre-treatment and purification of the starting materials are necessary. These methods typically include heating oxide precursors at high temperatures (1000 °C) to remove residual moisture and volatile species, as well as fluorinating methods prior to melting to further reduce oxide-related effects [2]. The following subsections present common glass preparation steps employed in research and industrial fluoride glasses production.

### 2.1. Purification of Raw Materials and Fluorination

To achieve high purity of fluoride glasses, several purification steps are employed before and during glass melting. Besides thermal dehydration of oxide precursors at elevated temperatures, fluorination is employed as an essential purification step that influences the properties and optical quality of the final glass product. Ammonium bifluoride (NH_4_HF_2_) is the most widely used fluorinating agent since it decomposes to release HF which efficiently reacts with oxide and OH^−^ impurities [30]. This chemical compound can also reduce oxygen concentrations in HMFGs to approximately 10 ppm, resulting in improved thermal stability by suppressing heterogeneous nucleation [31]. Nevertheless, its performance remains controversial. Previous studies demonstrated that excessive NH_4_HF_2_ additions produce residual ammonium species responsible for absorption bands around 2.9 μm [32], while Cimek et al. [33] observed that even when high-purity (5 N) ZrF_4_ was employed, NH_4_HF_2_ frequently resulted in grey-colored glasses containing black crystalline inclusions. These observations suggest that although ammonium bifluoride effectively removes oxygen contamination, it does not necessarily provide the highest optical quality required for modern low-loss fluoride fibers.

Xenon difluoride (XeF_2_) and sulfur hexafluoride (SF_6_) have emerged as alternative and effective fluorinating agents. Studies have shown that both compounds consistently produced optically transparent ZBLAN glasses when combined with high-purity zirconium tetrafluoride [33]. Their superior performance is attributed to maintaining a sufficiently high fluorine activity during melting, thereby suppressing the partial reduction of Zr^4+^ to Zr^3+^ and preventing the formation of black ZrF_3_ inclusions. Importantly, these results provide evidence that precursor purity alone is insufficient to guarantee high optical quality. Even ultra-high-purity (5 N) ZrF_4_ without fluorination still produced minor black impurities, whereas the combination of high-purity precursors with XeF_2_ or SF_6_ resulted in fully transparent glasses.

In summary, no fluorination strategy is universally superior. NH_4_HF_2_ remains attractive because of its low cost and widespread use, but it may introduce optical defects under certain processing conditions. In contrast, XeF_2_ and SF_6_ generally produce higher optical quality glasses, although their cost and environmental considerations limit widespread use. Therefore, the optimum fluorination strategy depends on the intended application, where optical performance, economic considerations and environmental impact must all be evaluated and balanced.

### 2.2. Moisture Control and Processing Atmosphere

Besides precursor purification, maintaining an extremely dry processing atmosphere is equally essential throughout fluoride glass fabrication. Unlike oxide glasses, fluoride melts react with atmospheric moisture, producing hydroxyl species and oxyfluorides that subsequently act as heterogeneous nucleation sites during cooling [28,29]. Consequently, HMFG synthesis is performed inside hermetically sealed gloveboxes under nitrogen or argon atmospheres, where residual oxygen and water concentrations are typically maintained below 5 ppm [34]. Moreover, such stringent environmental control is required not only during melting but also throughout casting, annealing, and even fiber drawing, since brief exposure to ambient humidity can introduce surface contamination and promote crystallization.

Several purification strategies have also been developed to remove residual impurities directly from the molten glass. Reactive atmosphere processing (RAP) employs reactive gases such as NF_3_ or CCl_4_ to convert oxide and hydroxyl species into volatile products that are subsequently removed from the melt [32,34]. Although both gases effectively reduce hydroxyl concentrations, NF_3_ is generally preferred because CCl_4_ may introduce carbon-containing impurities into the melt. However, RAP possesses only limited thermodynamic purification capability and cannot completely eliminate contaminants already present in the starting materials [35]. Consequently, reactive atmosphere processing should be regarded as a complementary purification technique rather than a substitute for high-purity precursors. These purification and moisture-control strategies are summarized in Table 1.

### 2.3. Melt-Quenching Technique

For a wide range of glass-forming systems, the melt-quenching technique remains the most established method for oxide glass synthesis and can be also utilized for the synthesis of HMFGs. This process includes the direct melting of high-purity fluoride precursors (e.g., ZrF_4_, BaF_2_, LaF_3_, AlF_3_, and NaF in the case of ZBLAN glass), which are accurately and homogeneously mixed with, utilizing automatic mortars to ensure fine particle size and compositional uniformity.

After purification of the raw materials, as discussed in Section 2.1, the prepared fluoride batch is transferred into platinum, or vitreous carbon crucibles for the melting step which is conducted at temperatures sufficiently above the liquidus temperature about 850 °C for ZrF_4_-based glasses, to ensure the rapid and complete dissolution of particles within the batch. A critical step in this process is the fining step, which involves heating the melt to high temperatures (above the liquidus) in an oxidizing atmosphere. This leads to glass homogenization without the need for mechanical stirring, eliminates volatile species, oxidizes and dissolves any residual phases that could cause light scattering, ultimately yielding a clear and homogeneous glass [5]. However, a challenge associated with high melting temperatures is the increased volatilization rates of certain fluoride compounds. In particular, ZrF_4_ which sublimates at approximately 900 °C, exhibits an appreciable vapor pressure above 600 °C [36]. This phenomenon leads to excessive ZrF_4_ evaporation losses, which can alter the final glass properties and composition, thereby compromising reproducibility. For this reason, prolonged melting times at high temperatures should be avoided.

### 2.4. Casting and Cooling

After the melting and fining steps, the melt must be rapidly cooled down to form an amorphous solid. This process typically involves casting the melt into a metallic mold to ensure a high cooling rate, a process known as quenching, which is essential for promoting glass formation and preventing unwanted crystallization. Brass molds are frequently employed for this purpose and are typically preheated to a temperature a few degrees below the glass transition temperature (T_g_) of the glass to prevent thermal shock and cracking. Common mold materials beyond brass include graphite and stainless steel, which are used in various glass casting applications for their durability and ability to impart specific shapes.

### 2.5. Thermal Annealing

After casting and rapid cooling, the glass might have developed internal mechanical stresses arising from non-uniform cooling and the volume difference between the solid and liquid states during solidification. To mitigate this issue and release these stresses, thermal annealing is commonly performed. Annealing involves reheating the formed glass to a temperature close to its glass transition temperature, holding it at this temperature for a specific duration to enable structural relaxation, and subsequently cooling it slowly to room temperature at a controlled rate. For ZBLAN based glasses, the annealing temperature ranges from 250 to 300 °C. Furthermore, it has to be noted that the annealing time depends on the volume of the bulk glass. Larger components usually require longer annealing times to ensure complete stress relief throughout the material For ZBLAN glass, an annealing time of 1 h at the glass transition temperature is common [37]. After holding ZBLAN glass at a specified temperature, it is then cooled down slowly from 280 °C to room temperature at a common rate of about 0.5 °C/min [33]. After holding ZBLAN glass at a specified temperature, it is then cooled down slowly from 280 °C to room temperature at a common rate of about 0.5 °C/min [33]. Another reported cooling rate is 0.3 °C/min [38]. This slow cooling is crucial to prevent the reintroduction of new thermal stresses [5]. Finally, once the glass cools below its strain point, the inherent permanent mechanical stress cannot be changed by further cooling rate adjustments.

## 3. Structure and Physical Properties of Fluorozirconate Glasses

### 3.1. Structural Characteristics of Fluorozirconate Glasses

Fluorozirconate glasses differ fundamentally from conventional oxide glasses since their atomic structure is governed by ionic bonds rather than highly covalent bonds. Consequently, these glasses consist of interconnected zirconium-fluorine polyhedra with various coordination numbers and connectivity, resulting in a more flexible glass network. Despite decades of investigation, a universally accepted structural model has not yet been established because the structural information obtained depends strongly on the experimental technique employed and the structural length scale being probed [5,39].

**Figure 1:**
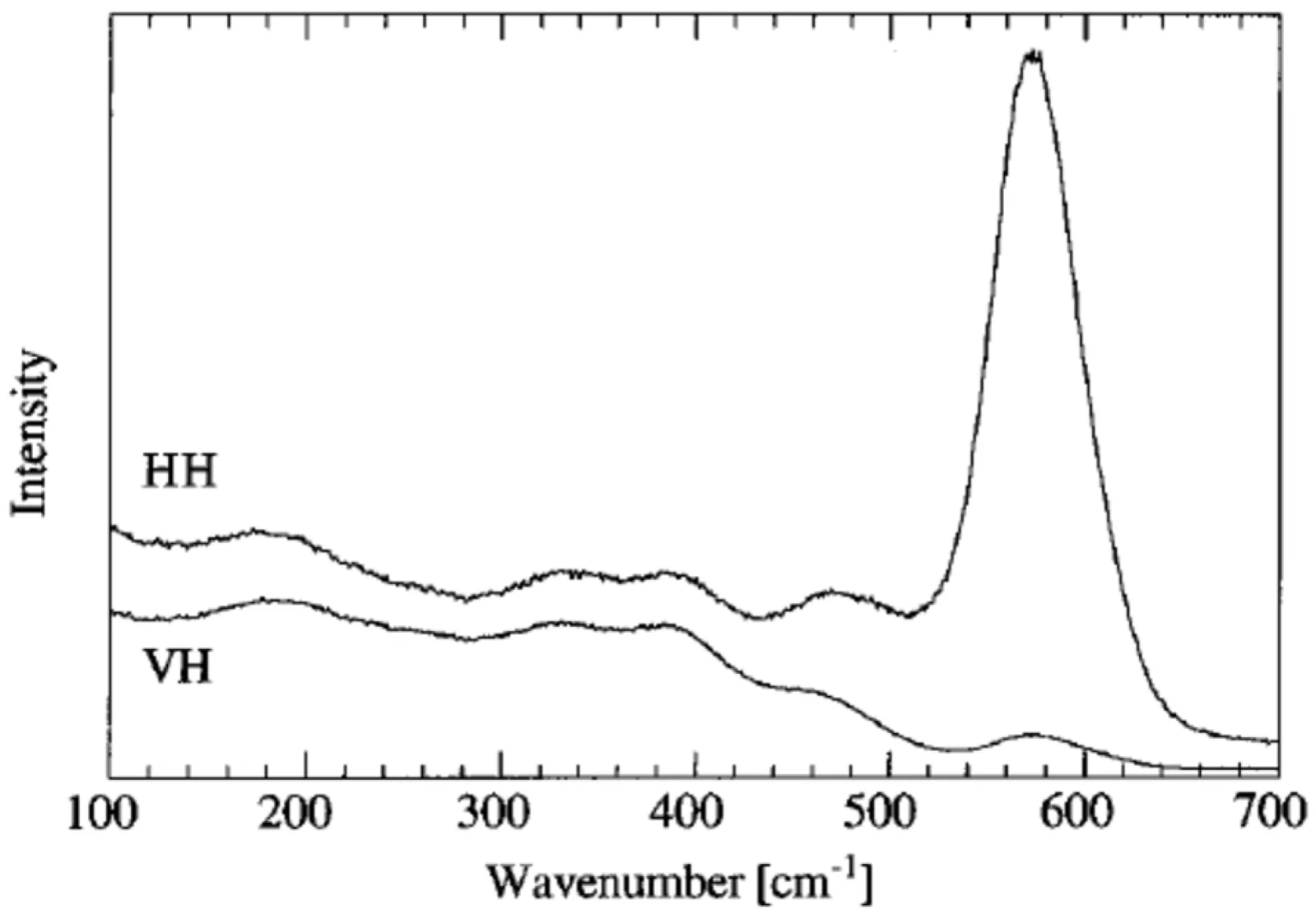
Polarized HH and depolarized Raman spectra of the glass 50ZrF_4_-25BaF_2_-25NaF. Reused with permission from Ref. [43].

The first structural models of fluorozirconate glasses were established mainly from Raman and IR spectroscopic data of binary and ternary ZrF_4_ based glass systems [39,40]. These studies suggested that the fluorozirconate glass network consists of interconnected ZrF_6_ octahedra linked through bridging fluorine atoms, while modifier cations (e.g., Ba^2+^, Na^+^, Li^+^) occupy interstitial sites and influence the connectivity of the network and the glass properties. Raman spectra of such glasses indicate that the variations of the F/Zr ratio modify the connectivity of the zirconium fluoride polyhedra through changes in the zirconium coordination number. Nevertheless, Raman spectroscopy alone cannot distinguish the difference in zirconium coordination number (e.g., six, seven or eight-fold) due to the complexity of the vibrational bands associated with these structural units which overlap. A typical Raman spectrum of the 50ZrF_4_-25BaF_2_-25NaF glass is displayed in Figure 1. The principal five Raman bands (565–598, 468–500, 386–416, 322–348, and 183–196 cm^−1^) are located and assigned to symmetric stretching vibrations of terminal fluorine bonds Zr–F_T_ within zirconium fluoride polyhedra asymmetric stretching modes and bending vibrations of Zr–F bonds [39,40,41,42,43].

Complementary structural information has subsequently been obtained from X-ray scattering, EXAFS and neutron diffraction measurements [40,44,45,46]. X-ray and neutron diffraction provide average pair-distribution functions and interatomic distances, whereas EXAFS yields element-specific information on the local environment surrounding zirconium and rare-earth ions. These studies consistently indicate that zirconium does not exhibit a specific coordination number but instead exists within a distribution of local structural configurations. However, both EXAFS and diffraction measurements represent ensemble averages over the entire glass structure and therefore cannot uniquely determine the intermediate range order of the network.

To overcome these limitations, modern structural investigations increasingly combine experimental spectroscopic and diffraction data with atomistic modelling approaches such as molecular dynamics (MD) simulations and Reverse Monte Carlo (RMC) [47,48]. RMC has become one of the most widely adopted modelling techniques for disordered materials because it simultaneously refines large atomistic configurations against diffraction and spectroscopic datasets while incorporating physically meaningful structural constraints [49]. Recent methodological developments in constrained RMC and total-scattering analysis have significantly improved the reliability of structural models for complex glasses and amorphous materials [49,50]. Nevertheless, only a limited number of studies have applied these approaches specifically to fluorozirconate or ZBLAN glasses, the available experimental and computational evidence consistently supports the coexistence of ZrF_6_, ZrF_7_ and ZrF_8_ polyhedra connected through both corner- and edge-sharing fluorine atoms, with their relative abundance depending on glass composition, fluorine activity during melting and thermal history [5,40]. This picture is also consistent with recent reviews emphasizing that the local structure of HMFGs is better described by a distribution of coordination environments [5].

**Figure 2:**
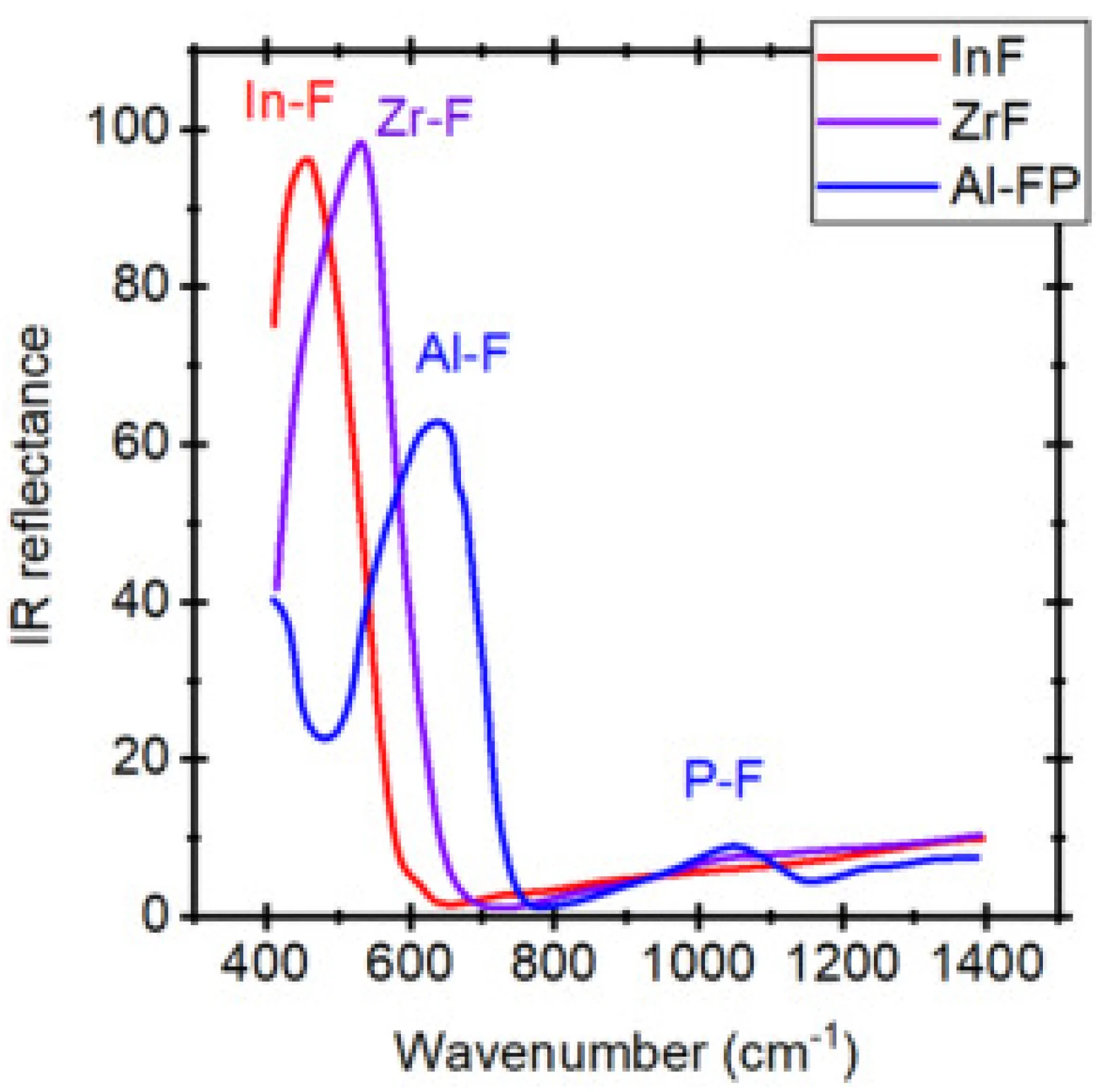
Infrared spectral shift with the mass of the cation. Reused under CC-BY-NC-ND license from Ref. [52].

Another important contribution to understanding the fluoride glass structure arises from the relationship between metal-fluorine (M-F) bond strength and vibrational frequency of typical fluoride glasses. A spectroscopic study has demonstrated that the frequencies of the M-F stretching vibrations decrease with increasing cation mass, while higher cation coordination numbers are also generally associated with lower stretching frequencies. Figure 2 illustrates the trend of the infrared spectral shift with increasing cation mass, based on the data reported in Ref. [51].

Overall, the current body of experimental and computational evidence indicates that fluorozirconate glasses should be regarded as structurally heterogeneous ionic networks rather than materials described by a single zirconium coordination number. Early Raman and infrared studies established the importance of ZrF_6_ based structural units, whereas subsequent diffraction, EXAFS and atomistic modelling demonstrated that zirconium coordination is distributed over several local environments whose relative populations depend on composition and processing conditions. Despite significant advances, comparatively very few investigations have combined neutron diffraction, EXAFS and constrained Reverse Monte Carlo refinement specifically for fluorozirconate and ZBLAN glasses, in contrast to the extensive literature available for oxide glasses. Future studies integrating high-energy scattering, neutron diffraction with modern constrained RMC and molecular dynamics simulations are therefore expected to significantly improve the understanding of intermediate range order and establish more quantitative structure property relationships for fluorozirconate glasses, thereby providing a stronger scientific basis for the design of next-generation fluoride photonic materials.

### 3.2. Thermal Properties

Heavy metal fluoride glasses (HMFGs) are generally considered soft glasses due to their viscoelastic behaviour and relatively low melting and processing temperatures (<1000 °C) compared with silicate and many other oxide glasses. Their thermal properties can be characterized by utilizing modern thermal analysis methods including simultaneous thermal analysis (STA), differential scanning calorimetry (DSC), and differential thermal analysis (DTA). These experimental methods are widely employed in glass science to detect phase transitions such as melting and crystallization, as well as to determine characteristic temperatures, including the glass transition temperature (T_g_), onset crystallization temperature (T_x_), and peak crystallization temperature (T_p_). One of the major drawbacks of most HMFGs is their tendency to crystallize when heated between the glass transition and melting temperatures. Consequently, only a few glass compositions exhibit stability to crystallization [5]. It should be noted that ZBLAN glass exhibits good thermal stability and can be readily drawn into optical fibers.

Fluorozirconate glasses can also incorporate HfF_4_ as a substitute, since hafnium has a similar atomic radius to zirconium atom but a higher atomic mass. It has been reported that the addition of hafnium does not significantly alter most physical properties of the glass. However, it may cause a decrease in the refractive index and an increase in the glass transition temperature [40]. In most fluorozirconate glasses, T_g_ typically ranges from 240 to 330 °C and is mainly influenced by the concentration of alkali metals and barium. Increasing the alkali fluoride content generally lowers the glass transition temperature because alkali ions act as network modifiers, reducing the connectivity of the Zr–F polyhedral network and facilitating atomic rearrangement during the glass transition [53]. In contrast, fluoroaluminate glasses may exhibit higher T_g_ values, often exceeding 35 °C. The alkali and barium content also contribute to an increase in the thermal expansion coefficient which is attributed to the replacement of rigid Al–F structural units by more weakly bonded ionic species, which reduce network rigidity, increase free volume, and permit greater thermal expansion [54]. An exception is the substitution of NaF with LiF, which results in a decrease in the thermal expansion coefficient. In this case, T_g_ also decreases [55]. Table 2 summarizes various characteristic temperatures and thermal properties of representative HMFGs, with an emphasis on fluorozirconate glass compositions [56,57,58,59,60].

The reported thermal properties of the glasses presented on Table 2 were determined using differential thermal analysis at a heating rate of 10 K/min. Based on the values of the glass transition temperarure (*T_g_*) and the onset of crystallization temperature (*T_x_*) it is evident that HMFGs exhibit a relatively narrow temperature range of thermal stability. This behaviour is reflected in the thermal stability parameter S which was calculated using relation (1). Thermal stability *S* (also expressed as Δ*T*) is a significant parameter, particularly for optical fiber drawing as it expresses the material’s resistance to thermal shock and is commonly determined by the relation [61]:

(1) $$S=T_{x}-T_{g}$$

Besides the thermal stability window (S), other parameters have been proposed to evaluate the crystallization resistance of glasses. One of the most widely used is the Hruby parameter, $K_{H}=(T_{x}-T_{g})/(T_{m}-T_{x})$, which incorporates the melting temperature and therefore provides a more comprehensive assessment of thermal stability [62]. In fluoride glasses, however, S remains the most commonly reported parameter because it is directly related to the processing window for fiber drawing, while Hruby’s parameter is reported less frequently due to the additional requirement of accurately determining the melting or liquidus temperature. In general, glasses with thermal stability values higher than 100 °C are considered to be of high quality with respect to their optical properties [63,64,65]. However, a well-known limitation of fluoride glasses is their crystallization tendency due to their low viscosity, and thus, only a limited number of compositions are stable. Another factor that limits the development and optimization of these glasses is the incomplete understanding of their structure-properties relationship. Hence, the design and investigation of new glass compositions remain an important issue for future research aimed at improving and tailoring the properties of HMFGs.

### 3.3. Mechanical Properties

HMFGs are generally classified as soft materials compared to conventional oxide glasses, such as silicate glasses, borosilicates etc. The predominance of ionic bonding in fluoride glasses leads to reduced network connectivity and structural rigidity compared to the strongly covalent and highly interconnected networks of oxide glasses, resulting in lower intrinsic mechanical strength, hardness and elastic moduli. In particular, the fracture toughness of fluoride glasses is relatively low, ranging between 0.25 and 0.30 MPa·m^1/2^, while their microhardness is reported in the range of 200–300 kg/mm^2^ [5]. These values are significantly lower than those of silicate glasses, reflecting the reduced resistance of fluoride glasses to cracks. However, the mechanical properties of HMFGs do not solely depend on their chemical composition. Extrinsic defects such as surface flaws, microcrystallites, as well as compositional inhomogeneities that might be introduced during glass synthesis and processing can also affect the measured mechanical properties. As a result, experimentally reported values often underestimate the intrinsic strength of the material, making direct comparison between different studies difficult.

The elastic behaviour of fluorozirconate glasses is commonly characterized by ultrasonic echography, in which longitudinal and transverse sound velocities are measured together with the glass density. These measurements enable the determination of the longitudinal, shear, bulk, and Young’s moduli, as well as Poisson’s ratio. Hardness is typically evaluated using Vickers or Knoop indentation methods. These techniques provide complementary information regarding the resistance of the glass network to elastic and plastic deformation. A detailed description of ultrasonic echography for such measurements can be found in the literature [66].

Representative elastic properties of fluorozirconate glasses are summarized in Table 3. The reported Young’s modulus typically lies between approximately 51 and 55 GPa, while the shear modulus varies between 20 and 22 GPa, indicating that moderate compositional modifications produce relatively small changes in network rigidity. Similar behaviour has been reported for other fluoride glass families, suggesting that their elastic response is fundamentally controlled by the ionic nature of the glass network rather than by minor compositional variations.

Table 4 compares the mechanical properties of the principal fluoride glass families with those of silicate glasses (data are based on glass fibers). Fluoroaluminate glasses generally exhibit the highest Young’s modulus and hardness among fluoride glasses owing to the stronger Al–F bonds and the higher field strength of Al^3+^, which increase network connectivity. By comparison, fluorozirconate glasses possess intermediate elastic properties but significantly better infrared transparency, while fluoroindate glasses generally exhibit the lowest stiffness. This comparison highlights one of the fundamental trade-offs in fluoride glass science: improvements in optical performance are frequently accompanied by reduced mechanical robustness.

Overall, the available literature indicates that the outstanding optical performance of fluorozirconate glasses is achieved at the expense of mechanical strength. Future research should therefore focus on developing compositions that enhance network rigidity without significantly increasing phonon energy or reducing infrared transparency. In particular, combining experimental mechanical characterization with atomistic modelling and structural analysis is expected to provide a deeper understanding of the composition–structure–property relationships governing the mechanical behaviour of heavy metal fluoride glasses in general.

### 3.4. Infrared Transmission of Heavy Metal Fluoride Glasses

HMFGs exhibit exceptional optical properties, originating from their structural characteristics and chemical composition. These glasses present large transparency bandwidth of optical transmission from the UV to the mid-infrared (MIR) spectral region making them suitable materials for several photonic applications. Their broad transparency in the UV and MIR is attributed to the low electronic polarizability of the constituent ions and the low fundamental resonance frequency of the metal-fluorine (M-F) bonds. As the energy of stretching vibrations between the metal and fluorine ions decreases, the multi-phonon absorption edge is shifted to longer wavelengths, extending the MIR transmission beyond that of common oxide glasses. Specifically, it has been reported that the energy of the stretching vibration decreases in the following order: AlF_3_ > ZrF_4_ > InF_3_ > MF_2_ [14]. The maximum phonon energy of HMFGs together with the UV and IR cutoff is shown in Table 5.

### 3.5. Refractive Index of Heavy Metal Fluoride Glasses

The refractive index of HMFGs is related to their chemical composition, structure, and the polarizability of $F^{-}$ anions and their heavy cations. The refractive index values of such glasses typically vary between 1.4 and 1.5. ZBLAN glass has a refractive index close to 1.5 which originates from the high polarizability of heavy metal cations and its open fluorozirconate network [68]. Other HMFGs such as fluoroaluminates exhibit a slightly lower refractive index about 1.4–1.5 attributed to the presence of the stronger Al–F bonds. Generally, glass compositional changes due to modifiers or dopants can alter the refractive index in various ways. For example, the addition of alkali fluoride modifies such as LiF can cause a decrease in the index, while PbF_2_ increases it.

The optical dispersion dn/dλ of HMFGs is negative in contrast to silicate glasses which exhibit a positive dispersion. Values of dn/dλ have been reported to range between −8 and −15 10^−6^/K [5]. Furthermore, the optical dispersion change is smaller compared to other glass categories such as chalcogenides [70].

## 4. Spectroscopic Properties of Er^3+^, Ho^3+^ and Tm^3+^ Doped Fluorozirconate Glasses

### 4.1. Thermal Properties of Er^3+^ Doped Fluorozirconate Glasses

Er^3+^ doped fluorozirconate glasses present scientific and technological interest for mid-infrared applications in laser technology due to their spectroscopic properties. In particular, their characteristic emissions at approximately 1.5 μm and 2.7–2.8 μm, originating from the ^4^I_13/2_ → ^4^I_15/2_ and ^4^I_11/2_ → ^4^I_13/2_ transitions, respectively, make Er^3+^ doped fluorozirconate glasses attractive materials for optical communication, eye-safe lasers, medical surgery, environmental monitoring and infrared measure systems [16,71,72]. Unlike oxide glasses, their lower phonon energy (~500–600 cm^−1^) considerably suppresses multiphonon relaxation, thereby increasing the probability of radiative transitions and enabling efficient emission around 2.7 μm, which is strongly quenched in silicate hosts. Consequently, several investigations have focused not only on the spectroscopic characteristics of Er^3+^ ions, but also on the thermal stability of the host glass, since both properties determine the feasibility of fabricating low-loss optical fibers.

**Figure 3:**
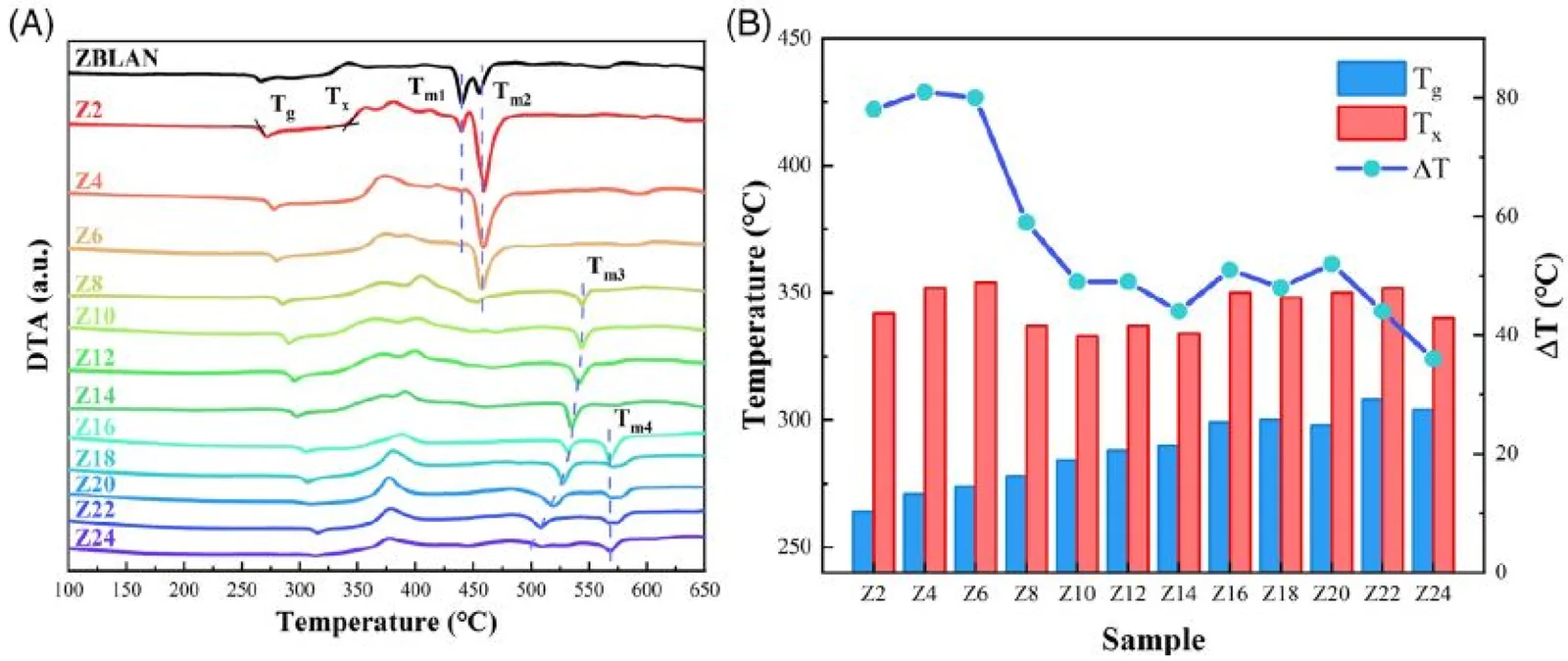
(**A**) Thermographs of Er^3+^ doped ZBLAN glasses. (**B**) Graph of T_g_, T_x_ temperatures and thermal stability for Z1–Z25 glasses. Reused with permission from Ref. [73].

A comprehensive study of the effect of Er^3+^ concentration on the thermal properties of ZBLAN glass was reported by Liu et al. [73] who synthesized and studied the glass composition 53ZrF_4_-20BaF_2_-4LaF-3AlF_3_-20NaF-xErF_3_ with x ranging from 1–25 mol% (designated as Z1–Z25 glasses). Their results demonstrated that increasing Er^3+^ concentration increases the glass transition temperature, while simultaneously reducing the thermal stability as shown in Figure 3B. The increase in the glass transition temperature with increasing Er^3+^ concentration is generally attributed to the structural role of Er^3+^ ions in the fluorozirconate glass network. Due to their relatively high ionic field strength, Er^3+^ ions form stronger Er–F interactions than the modifier cations they replace (e.g., La^3+^), leading to a more compact and rigid local structure [73]. This enhanced structural rigidity reduces atomic mobility and shifts the glass transition to higher temperatures, although the magnitude of the effect depends on the glass composition and the Er^3+^ concentration.

Although the work of Liu et al. [73] provides a systematic investigation of the Er^3+^ effect on glass thermal behaviour, other researchers have demonstrated that partial substitution of NaF by LiF influences crystallization and thermal behaviour without substantially altering the characteristic spectroscopic properties of Er^3+^ doped ZBLAN glasses Yuan et al. [74]. Their study showed that low to moderate LiF concentrations improved the thermal stability parameter, indicating enhanced resistance to crystallization while preserving the favourable optical characteristics of the fluoride host glass composition.

Taken together, these studies demonstrate that the thermal behaviour of Er^3+^ doped fluorozirconate glasses is governed by both rare-earth concentration and host-glass composition [5]. Increasing the Er^3+^ concentration generally enhances network rigidity, leading to higher glass transition temperatures, but excessive doping also promotes crystallization and reduces thermal stability. In contrast, compositional modifications through the mixed-alkali effect improve glass-forming ability without substantially altering the glass transition temperature. These findings indicate that optimizing Er^3+^ doped ZBLAN glasses requires balancing dopant concentration with compositional design, rather than maximizing either parameter independently. However, the mechanisms by which rare-earth ions simultaneously increase network rigidity while reducing resistance to crystallization remain insufficiently understood. Clarifying this relationship represents an important direction for future studies aimed at improving the thermal performance of rare-earth doped fluorozirconate glasses in general.

### 4.2. Spectroscopic Properties of Er^3+^-Doped Fluorozirconate Glasses

Er^3+^ doped ZBLAN glass remains one of the most efficient fluoride hosts around 2.7 to 2.8 μm [72]. Studies have also reported spontaneous emission probabilities for the ^4^I_11/2_ → ^4^I_13/2_ transition [16,75]. The Er^3+^ concentration seems to influence glass spectroscopic behaviour since it has been reported that increasing erbium concentration increases the absorption coefficient thereby improving pump absorption, particularly under excitation near 980 nm [22,73,75]. However, this improvement is only observed up to an optimum concentration. Beyond this point, the average separation between neighbouring Er^3+^ ions decreases sufficiently for ion–ion interactions to become significant. Energy-transfer upconversion (ETU), excited-state absorption (ESA), and cross-relaxation processes then become increasingly probable, reducing the population of the upper laser level and shortening the fluorescence lifetime [20,71,75]. As a result, the increase in pump absorption is accompanied by a reduction in quantum efficiency, illustrating that higher rare-earth concentrations do not necessarily lead to improved laser performance. Consequently, the optimum Er^3+^ concentration must be selected based on both spectroscopic and thermal stability parameters, since sufficient thermal stability is also important for drawing these glasses into fibers.

**Figure 4:**
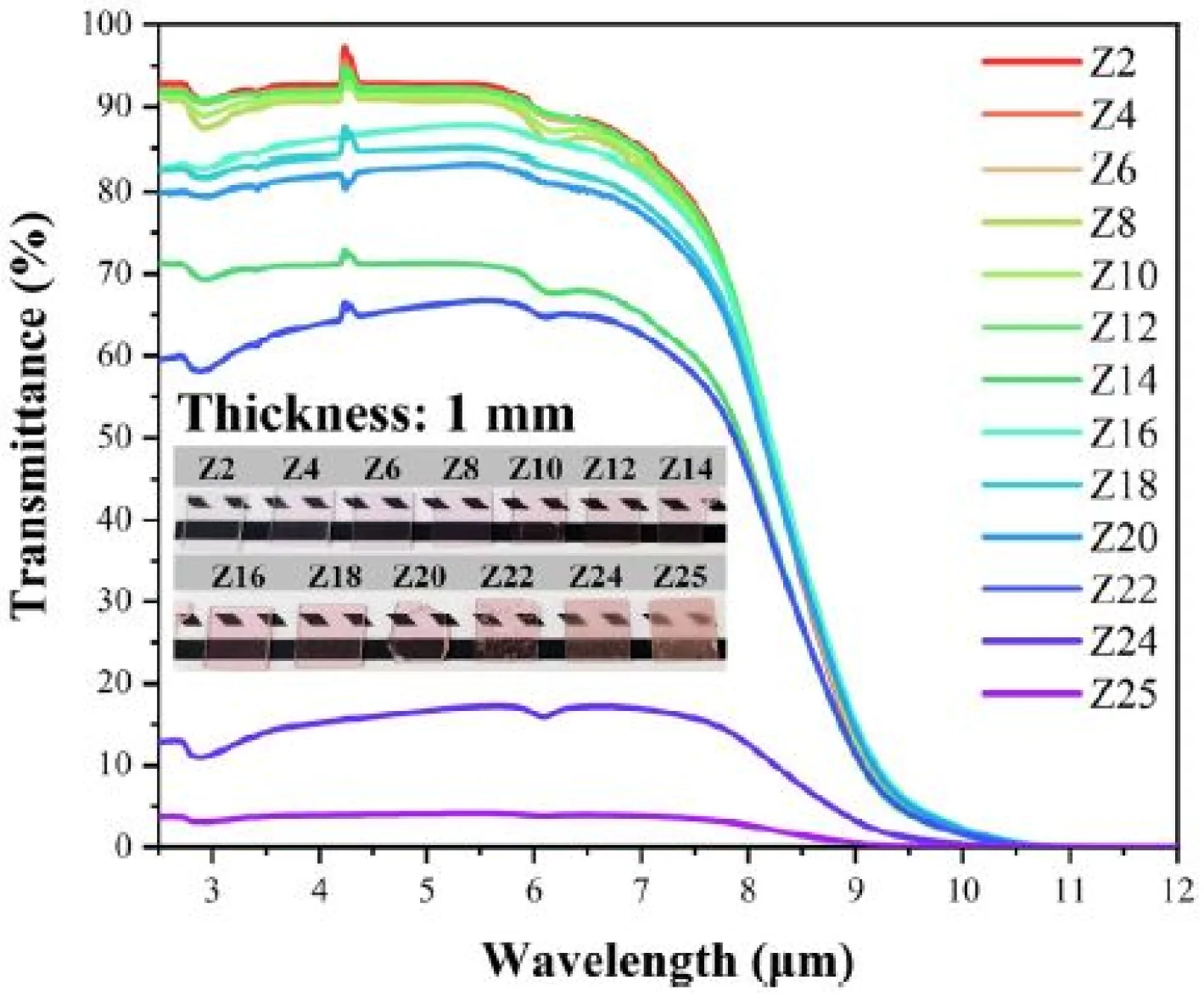
IR transmittance of ZBLAN glasses doped with different Er^3+^ concentrations. Reused with permission from Ref. [73].

The transmittance of Er^3+^ doped ZBLAN glasses is also affected by Er^3+^ concentration [54]. It has been reported that for low Er^3+^ doping (Z2–Z12 glasses), the transmittance is generally high ~90% but decreases with further increase in Er^3+^ concentration (see Figure 4). Moreover, for good infrared transmittance the suitable Er^3+^ doping concentration is up to 7 mol%.

The emission intensity at 1.5 μm increases with Er^3+^ concentration up to approximately 10–12 mol%, reflecting the higher density of optically active ions and the corresponding increase in pump absorption efficiency. Beyond this concentration, however, the fluorescence intensity decreases because the average distance between neighbouring Er^3+^ ions becomes sufficiently small to promote non-radiative energy-transfer processes, including cross-relaxation and energy-transfer upconversion. These processes depopulate the emitting levels and reduce the radiative efficiency, resulting in concentration quenching. Consequently, although higher Er^3+^ concentrations enhance optical absorption, they do not necessarily improve laser performance, indicating that an optimum dopant concentration of approximately 11 mol% exists for efficient 2.7 μm emission in ZBLAN glasses [54].

**Figure 5:**
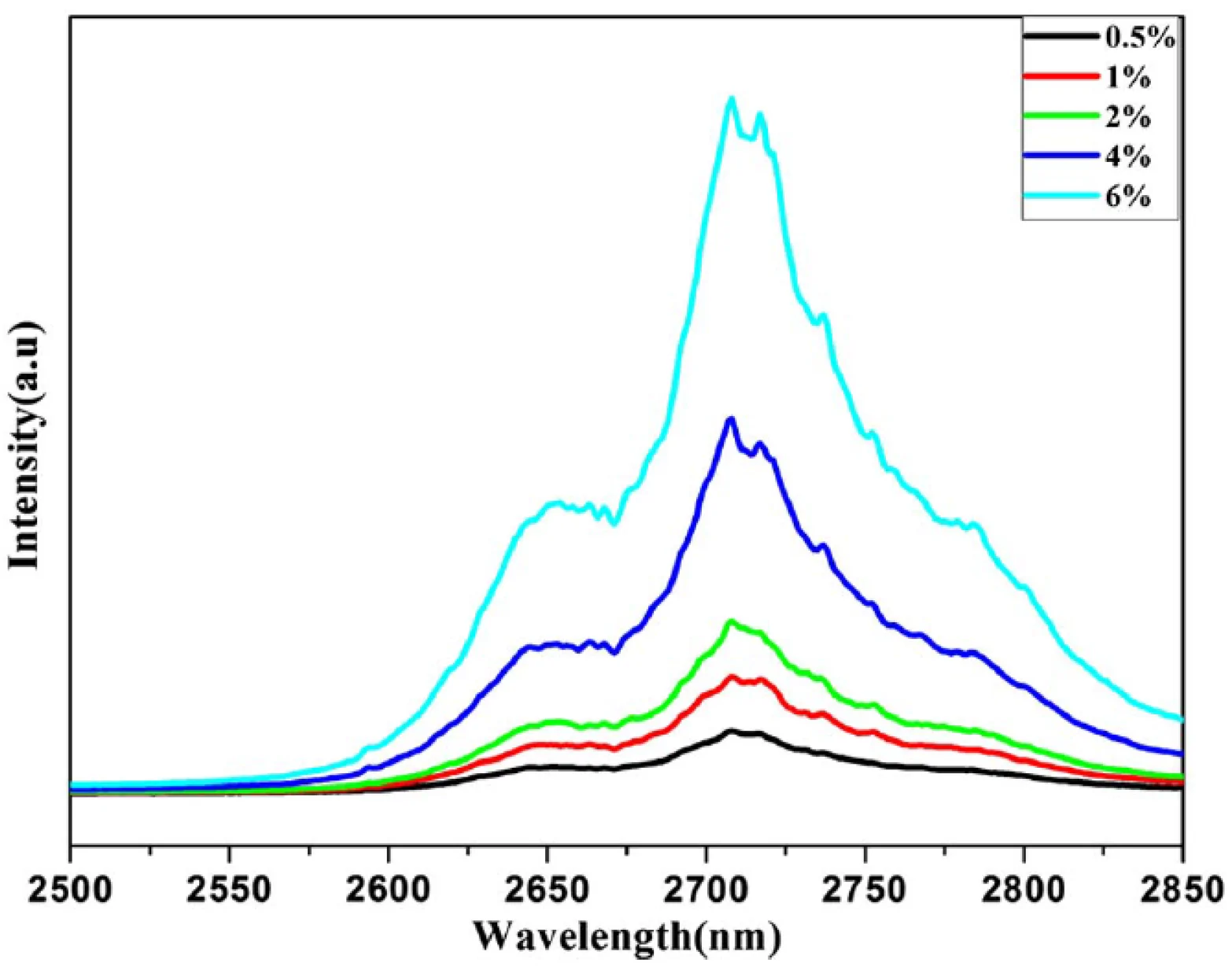
Emission spectra of Er^3+^ doped ZBYA glasses up to 6 mol% Er^3+^. Reused with permission from Ref. [75].

The concentration-dependent behaviour observed in ZBLAN glasses is not unique to this fluorozirconate composition. Similar trends have been reported for other fluorozirconate glass compositions including 50ZrF_4_-33BaF_2_-(AlF_3_ + YF_3_)-xErF_3_ (ZBYA), where Huang et al. [75] observed maximum fluorescence intensity near 2.7 μm for approximately 6 mol% ErF_3_ under 980 nm excitation. Although the optimum Er^3+^ concentration differs between ZBLAN and ZBYA, both systems exhibit the same fundamental behaviour: fluorescence intensity initially increases as the number of optically active centers rises but subsequently decreases because of concentration quenching. The different optimum concentrations are believed to originate from differences in the local coordination environment and the average Er–Er separation, which influence the probability of non-radiative energy-transfer processes. These observations demonstrate that the optimum Er^3+^ concentration is strongly host-dependent and reflects the interplay between glass composition, local rare-earth coordination and concentration-dependent energy-transfer processes. Consequently, the optimum dopant concentration cannot be generalized across different fluoride glass systems. The corresponding emission spectra are shown in Figure 5.

### 4.3. Spectroscopic Properties of Ho^3+^ Doped ZBLAN Glasses

Holmium (Ho^3+^) doped ZBLAN glasses have been studied and used for the fabrication of Ho^3+^ doped ZBLAN optical fibers as high gain media for lasers emitting at 1.2, 2, 2.86, 3.22 and 3.9 μm [57,58,59,61,62]. Various studies have also investigated theoretically the lasing characteristics of Ho^3+^ doped fluoride glasses in general [76,77,78].

**Figure 6:**
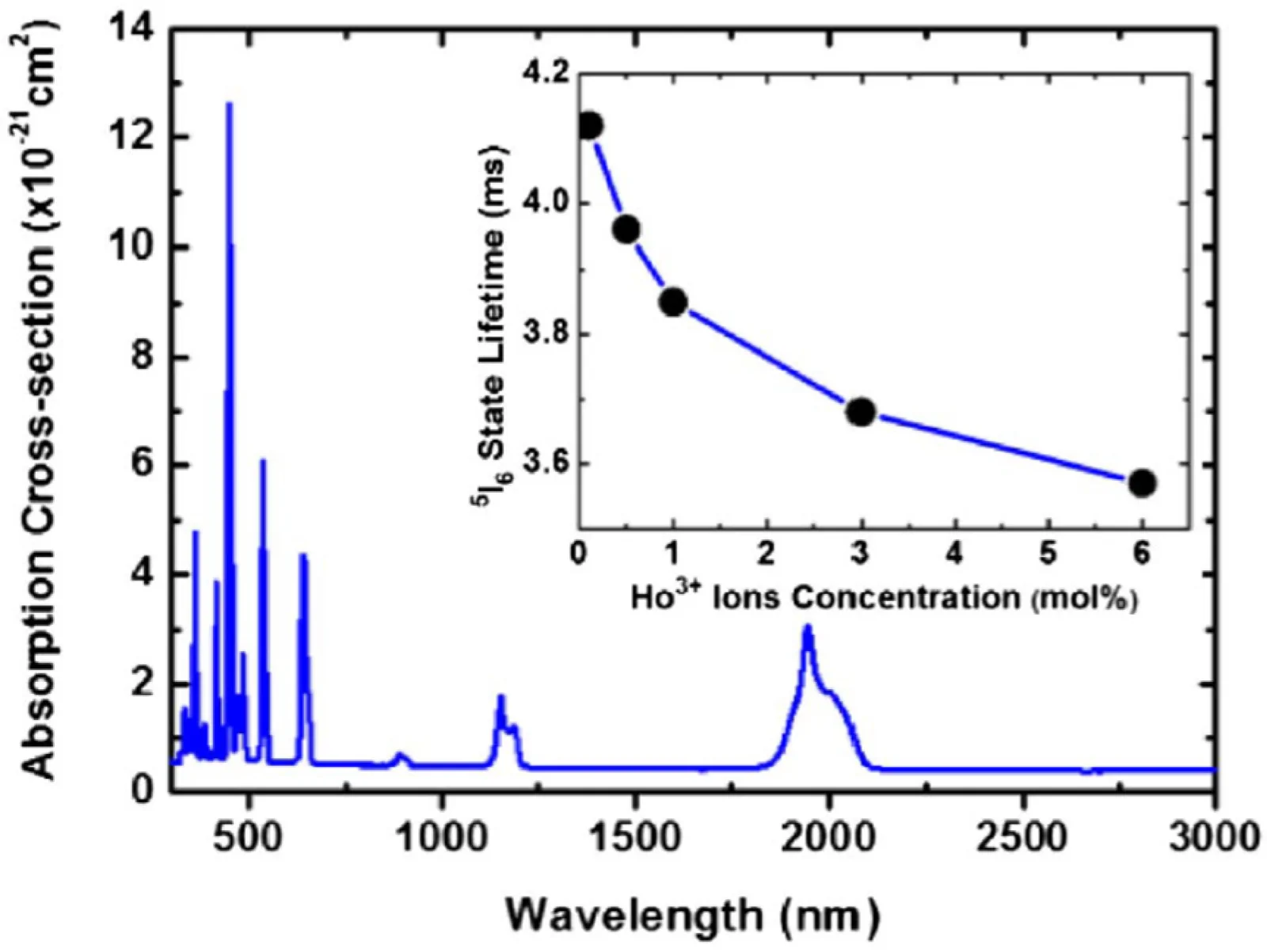
Absorption cross section and fluorescence lifetimes for Ho^3+^ doped ZBLAN glasses at different Ho^3+^ concentrations. Reused with permission from Ref. [23].

Except for its strong absorption in the visible spectrum, Ho^3+^ also presents an absorption at ~1150 nm. This wavelength is used to pump and study the emission of Ho^3+^ doped ZBLAN glasses. When Ho^3+^ doped ZBLAN glass is pumped by at 1150 nm, the upper laser level ^5^I_6_ is populated. Then, the transition from the excited energy state ^5^I_6_ to the ground state ^5^I_8_ causes emission at 1.2 μm, whereas the transitions ^5^I_7_ → ^5^I_8_ and ^5^I_6_ → ^5^I_7_ generate emissions at 2.1 μm and 2.9 μm respectively [19]. Furthermore, different concentrations of Ho^3+^ doped ZBLAN glasses have been prepared to study the concentration effect of Ho^3+^ on ZBLAN glasses on fluorescence and the lifetimes of the upper energy level ^5^I_6_. As shown in Figure 6 [23].

Figure 6 illustrates the fluorescence spectra of Ho^3+^ doped ZBLAN glasses with different Ho^3+^ concentrations. The characteristic emission bands arise from the ^5^I_6_ → ^5^I_8_, ^5^I_7_ → ^5^I_8_, and ^5^I_6_ → ^5^I_7_ transitions. As shown in the figure, variations in Ho^3+^ concentration modify the relative fluorescence intensities of these transitions, reflecting changes in the population of the excited energy levels. The observed behaviour demonstrates that, although increasing the Ho^3+^ concentration increases the number of optically active centres, optimization of the dopant concentration remains essential for obtaining efficient infrared emission. Similar spectroscopic characteristics have been reported for Ho^3+^-doped ZBLAN glasses by [77], who measured the absorption spectra, fluorescence spectra, radiative lifetimes and Judd–Ofelt parameters for several rare-earth ions incorporated into the ZBLAN host. Their study demonstrated that the low-phonon fluoride environment provides relatively high quantum efficiencies and long radiative lifetimes by reducing multiphonon relaxation compared with oxide hosts, thereby confirming ZBLAN as an efficient host material for Ho^3+^ infrared laser transitions.

While the study discussed above focuses primarily on the spectroscopic response of Ho^3+^ ions, later work has shown that the performance of Ho^3+^-activated fluorozirconate glasses is also strongly influenced by the thermal stability of the host matrix. Ebendorff-Heidepriem et al. [79] investigated Ho^3+^ doped ZBYA fluorozirconate glasses and correlated their absorption and fluorescence properties with crystallization behaviour and thermal characteristics. Their results demonstrated that Ho^3+^ incorporation affects not only the optical response but also the nucleation behaviour of the fluoride glass. At relatively low Ho^3+^ concentrations the glass exhibited improved resistance to spontaneous nucleation, whereas higher Ho^3+^ concentrations promoted heterogeneous crystallization and increased the nucleation rate. These observations highlight that the optimization of Ho^3+^-activated fluorozirconate glasses requires simultaneous consideration of spectroscopic efficiency and thermal stability, since crystallization-induced heterogeneities can degrade optical quality and increase scattering losses.

Overall, the available literature demonstrates that Ho^3+^ doped fluorozirconate glasses remain among the most promising gain media for laser emission in the 2–3 μm spectral region because they combine favourable spectroscopic properties with the low phonon energy and broad infrared transparency of fluoride hosts. Nevertheless, compared with Er^3+^-doped fluorozirconate glasses, relatively few systematic investigations have correlated glass composition, local structure and processing conditions with the spectroscopic behaviour of Ho^3+^ ions. Recent reviews have also emphasized that further progress in fluoride laser materials requires improved understanding of the relationships between glass composition, structural stability and rare-earth ion environments [5]. Future studies combining advanced structural characterization, thermal analysis and spectroscopic modelling are therefore expected to facilitate the design of fluorozirconate glasses with improved efficiency and long-term stability for mid-infrared laser applications.

### 4.4. Emission Properties of Tm^3+^ Doped ZBLAN Glasses

Tm^3+^ doped fluoride glasses present spectroscopic properties which make them suitable materials for laser technology applications in the mid-IR. Among available hosts, ZBLAN and similar glasses such as ZBYA have demonstrated their superior spectroscopic performance, particularly in the 1.8–2.3 spectral region. For Tm^3+^ doped ZBLAN glasses around 2–3 mol%, the radiative transition of interest is the ^3^F_4_ → ^3^H_6_, yielding high gain, infrared radiation in the 1.8–2 μm spectral region [80]. The broad emission characteristics of such glass systems have been reported to arise from the inhomogeneous distribution of Tm^3+^ ions in the glass network [24,80]. Regarding Tm^3+^ doped ZBLAN glasses, it has also been reported that upon a 790 nm pump, the suppressed phonon environment of ZBLAN, this state is not instantly quenched by non-radiative decay, allows a 1.45 nm emission through the intermediate ^3^H_4_ → ^3^F_4_ transition [80]. In addition to the 1.8 μm emission, Tm^3+^-doped ZBYA glasses exhibit a strong dual band emission at 1.8 and 2.3 μm under excitation at 800 nm, with the 1.8 μm transition dominating due to efficient cross-relaxation processes that populate the ^3^F_4_ level [81]. Regarding fluorescence lifetime measurements, typical lifetimes of the ^3^F_4_ level in such glass-forming systems, are within ms range, which is much stronger than common oxide glasses due to reduced multiphonon relaxation. Furthermore, the relatively low phonon energy of fluoride glass hosts suppresses non-radiative decay pathways, enabling transitions, especially in the 2–3 μm region [4].

Although the long fluorescence lifetime of the ^3^F_4_ level is generally regarded as one of the principal advantages of Tm^3+^ doped fluoride glasses, laser performance is determined by several interdependent factors rather than lifetime alone. In particular, emission cross-section, pump absorption efficiency, cross-relaxation processes and the thermal stability of the host glass collectively influence laser efficiency. Consequently, optimization of Tm^3+^ fluorozirconate glasses requires balancing spectroscopic performance with glass-forming ability and thermal stability rather than optimizing a single material parameter.

Tm^3+^ doped ZBLAN glasses also exhibit upconversion luminescence, with blue emission bands observed around 450–480 nm under visible excitation, arising from excited-state absorption (ESA) and energy transfer mechanisms, with intensity scaling quadratically with pump power. However, it has been reported that concentration-dependent quenching effects become significant above ~0.2 mol% Tm^3+^, where cross-relaxation and clustering reduce upconversion efficiency [82].

Recent studies on co-doped glass systems further demonstrate the versatility of Tm^3+^ in fluoride glass hosts. For instance, Er^3+^/Tm^3+^ co-doped ZBLAN fibers exhibit broadband emission in the range of 1.4–2.0 μm and extending to ~2.7 μm, depending on excitation conditions. Moreover, emission bands between 1850 and 1980 nm, as well as 2625–2750 nm have been reported, indicating the potential of such doped glasses for multi-band amplification and tunable mid-IR sources [83]. Other co-doped fluoride glasses, such as Dy^3+^/Tm^3+^-doped ZrF_4_-BaF_2_-YF_3_-AlF_3_ systems, have also demonstrated efficient mid-infrared laser emission around 2.9 μm [84].

From a glass science perspective, modifications of the ZBLAN system, such as the transition to ZBYA compositions, have yielded measurable improvements in spectroscopic performance. Enhanced thermal stability reduces crystallization-induced scattering losses, while improved chemical durability minimizes OH^−^ contamination, one of the primary quenching centers in fluoride glasses. The reduction of hydroxyl absorption is particularly critical, as it directly influences mid-IR emission efficiency and lifetime [70].

In summary, these experimental studies suggest that Tm^3+^ doped ZBLAN and ZBYA glasses exhibit a combination of high emission cross-sections, long fluorescence lifetimes, broad emission bandwidths, and efficient cross-relaxation mechanisms. Such properties indicate that Tm^3+^ doped fluoride glasses can be promising laser glass materials in the 2 μm spectral region. Table 6 shows the main spectroscopic characteristics of the rare-earth doped glasses we discussed as follows.

## 5. Conclusions

This review has provided a comprehensive overview of the synthesis, structural, thermal, mechanical, optical and spectroscopical properties of fluorozirconate and rare-earth doped ZBLAN glass systems. Although the relatively low glass transition temperature of ZBLAN and related fluorozirconate glasses limits their use in applications requiring high thermal stability and high-temperature operation, it may also provide opportunities for emerging functional glass technologies. As demonstrated for low T_g_ oxide glasses, reduced processing temperatures can facilitate the incorporation of functional additives and the fabrication of glass-based composites [85]. Exploring similar strategies in fluorozirconate glasses may represent an interesting direction for future research, provided that their optical transparency, chemical durability, and crystallization resistance are preserved. Furthermore, these glasses exhibit lower mechanical strength, fracture toughness and elastic moduli than most oxide glass-forming systems. Due to their low phonon energies, such glasses exhibit broad transparency window extending from the UV to the mid-IR, and present exceptional solubility for rare-earth ions, making them suitable host materials for solid-state lasers and mid-infrared fiber optics.

One of the main conclusions emerging from this review is that the performance of fluorozirconate glasses is governed by a strong dependence between synthesis conditions, glass structure, and functional properties. Glass purity, fluorination strategy, moisture control, and thermal processing directly influence crystallization tendency and defect formation, which in turn determine optical transparency, thermal stability, etc. Consequently, optimization of glass processing conditions is equally as important as compositional design for achieving high-quality HMFGs.

The available structural studies indicate that fluorozirconate glasses cannot be described by a single structural model. Instead, experimental spectroscopy, diffraction techniques, and atomistic simulations consistently support the existence of heterogeneous networks composed of various zirconium coordination numbers. Despite considerable progress, quantitative structure–property relationships remain insufficiently understood because only a very limited number of studies have combined advanced experimental characterization with modern computational modelling.

Rare-earth doping with Er^3+^, Ho^3+^, and Tm^3+^ cations, enhances the properties of fluorozirconate glasses by enabling emissions in the near and mid-IR spectral regions. The dopant concentration plays an important role in determining their optical performance and even glass stability. Low to moderate dopant concentration may improve the glass spectroscopic properties, but high concentrations can reduce optical transparency and increase losses due to scattering. For example, in this article we report literature studies showing that Er^3+^ provides efficient emission around 2.7–2.8 μm, Ho^3+^ enables laser operation extending toward 3 μm and beyond, whereas Tm^3+^ is particularly attractive for broadband emission around 2 μm and efficient cross-relaxation processes.

In summary, fluorozirconate and ZBLAN based glasses continue to be used in mid-IR applications for their unique physical properties. However, progress and improvements in glass properties also need to be made. We believe that any progress in this field will come from a deeper understanding of their structure-properties relationships and overcoming their practical limitations. Future research should involve improved glass compositions and processing methods, as well as extended structural characterization utilizing modern spectroscopic techniques in combination with molecular dynamic modelling, machine learning algorithms and artificial intelligence. A predictive understanding of these structure–property relationships will enable the design of fluoride glasses with optimized structural rigidity and physicochemical properties.

## Figures and Tables

**Table 1:** Comparison of purification and moisture-control strategies used during HMFGs synthesis.

Purification Strategy	Purpose	Advantages	Limitations	References
NH_4_HF_2_	Removes OH^−^/O^2−^ by releasing HF	Widely used, cheap, reduces oxygen contamination	Residual NH4 species, may generate grey glass and black inclusions	[32,33,34]
XeF_2_	High efficiency fluorination	Produces transparent glass of excellent optical quality	Expensive	[33]
SF_6_	High fluorine activity during melting	Produces clear high quality transparent glass	Environmental concerns	[33]
RAP	Removes residual OH^−^ and O^2−^ from melt	Additional purification during melting	Limited purification.	[32,35]
Inert atmosphere (N_2_, Ar) in glovebox)	Moisture and oxygen contamination control	Essential for minimizing OH^−^ and oxyfluorides	Specific lab equipment (<5 ppm H_2_O/O_2_)	[28,29]

**Table 2:** Thermal properties of common fluorozirconate glass compositions (in mol%).

Glass Composition	T_g_ (°C)	T_x_ (°C)	S (°C)	α (10^−7^/K)	Ref.
57ZrF_4_-34BaF_2_-5LaF_3_-3AlF_3_	320	392	72	168	[56]
52ZrF_4_-24BaF_2_-4AlF_3_-20NaF	255	340	85	200	[56]
53ZrF_4_-20BaF_2_-4LaF-3AlF_3_-20NaF	263	370	107	200	[57]
53ZrF_4_-20BaF_2_-4LaF-3AlF_3_-20LiF	254	369	115	202	[60]
48ZrF_4_-17BaF_2_-4LaF_3_-3AlF_3_-20NaF-8PbF_2_	251	369	118	200	[58]
48ZrF_4_-17BaF_2_-4LaF_3_-3AlF_3_-20LiF-8PbF_2_	245	352	107	202	[58]
57HfF_4_-36BaF_2_-3LaF-4AlF_3_	325	426	101	165	[59]

**Table 3:** Elastic properties of 57ZrF_4_-(28.1 − x) BaF_2_-3.3LaF_3_-5AlF_3_-(6.6 + x) NaF (in mol%). Data reproduced with permission from Ref. [67].

E (±2 GPa)	G (±1 GPa)	K (±3 GPa)	σ	Hv (±10 kg/mm^2^)	NaF
53.3	20.5	44.4	0.299	-	7.6
51.2	19.9	40	0.286	213	8.6
53.7	20.7	43.6	0.290	195	9.6
54.6	21.8	46.5	0.310	208	10.6
54.3	21	43.3	0.290	180	11.6
55	21.7	39.1	0.270	198	16.6
55	21.3	43.8	0.290	220	20

**Table 4:** Mechanical properties of various fluoride fiber glasses. Data reproduced from Ref. [5].

Glass Type	E (GPa)	G (GPa)	K (GPa)	σ	H_k_ (kg·mm^−2^)
AFG	65	25	57	0.31	300–330
ZFG	52–55	19–21	40–46	0.29–0.32	190–250
IFG	44–55	19–21	47–52	0.31–0.33	180–230
Silicate	73	3100	36.7	0.17	770

**Table 5:** Comparison between the maximum phonon energy of heavy metal fluoride glass categories. Data based from [5,68,69].

Glass Category	Maximum Phonon Energy (${cm}^{-1}$)	IR Cutoff (μm)	UV Cutoff (nm)
Fluoroaluminates	600–650	7–8	160–180
Fluoroindates	480–520	9–10	250–300
Fluorozirconates	550–600	8–9	220–250

**Table 6:** Comparison of spectroscopic properties between Er^3+^, Ho^3+^, and Tm^3+^ doped fluorozirconate (ZBLAN) glasses.

Dopant	Main Emission Wavelength (μm)	Main Transition	Wavelength Pump (nm)
Er^3+^	1.53, 2.7–2.8	^4^I_13_/_2_ → ^4^I_15_/_2_, ^4^I_11_/_2_ → ^4^I_13_/_2_	980 (also 800 nm in some studies)
Ho^3+^	1.2, 2.1, 2.86–2.9, 3.9	^5^I_6_ → ^5^I_8_, ^5^I_7_ → ^5^I_8_; ^5^I_6_ → ^5^I_7_	1150
Tm^3+^	1.8–2.0, 2.3	^3^F_4_ → ^3^H_6_, ^3^H_4_ → ^3^H_5_	~790–800

## Data Availability

No new data were created or analyzed in this study. Data sharing is not applicable to this article.
